# Multimodal Imaging Analysis Reveals Frontal-Associated Networks in Relation to Individual Resilience Strength

**DOI:** 10.3390/ijerph18031123

**Published:** 2021-01-27

**Authors:** Shulan Hsieh, Zai-Fu Yao, Meng-Heng Yang

**Affiliations:** 1 CASE Lab, Department of Psychology, National Cheng Kung University, No.1, University Road, Tainan 701, Taiwan; reader2714@hotmail.com; 2 Institute of Allied Health Sciences, National Cheng Kung University, Tainan 701, Taiwan; 3 Department and Institute of Public Health, National Cheng Kung University, Tainan 701, Taiwan; 4 Brain and Cognition, Department of Psychology, University of Amsterdam, 1001 NK Amsterdam, The Netherlands; zaifuyao@gmail.com

**Keywords:** multimodal imaging, MRI, psychological resilience, personal strength, prefrontal cortex

## Abstract

Psychological resilience is regarded as a critical protective factor for preventing the development of mental illness from experienced adverse events. Personal strength is one key element of resilience that reflects an individual’s reactions to negative life events and is crucial for successful adaptation. Previous studies have linked unimodal imaging measures with resilience. However, applying multimodal imaging measures could provide comprehensive organization information at the system level to examine whether an individual’s resilience strength is reflected in the brain’s structural and functional network. In this study, MRI was used to acquire multimodal imaging properties and subscales of personal strength in terms of resilience from 109 participants (48 females and 61 males). We employed a method of fusion independent component analysis to link the association between multimodal imaging components and personal strength of psychological resilience. The results reveal that a fusion component involving multimodal frontal networks in connecting with the parietal, occipital, and temporal regions is associated with the resilience score for personal strength. A multiple regression model further explains the predictive role of frontal-associated regions that cover a visual-related network regulating cognition and emotion to discern the perceived adverse experience. Overall, this study suggests that frontal-associated regions are related to individual resilience strength.

## 1. Introduction

Psychological resilience is an important characteristic that can predict one’s success in every aspect of life and is considered to correlate with one’s mental wellbeing [1]. Some researchers have proposed that this characteristic involves numerous factors such as genetic, epigenetic, brain structure, brain function, neurochemical, physiological, developmental, demographic, cultural, economic, social, and psychological variables [2,3,4,5,6,7]. While all these variables play some roles in predicting the strength of psychological resilience, this study specifically focused on the aspects of brain structure and function in relation to the personal strength of resilience. This is because the main purpose of this study was to evaluate the brain model of psychological resilience. Although psychological resilience has drawn a lot of attention from researchers, limited work has used the neuroimaging approach to explore the neural correlates of resilience in a healthy population [3]. For example, a recent study by Kong et al. (2018) used blood-oxygen-level-dependent (BOLD) response to investigate the association between brain activation during resting and its relation to resilience [8]. Despite this study providing the first link between the functional organization of the brain and its role in resilience, whether these reported brain regions are generally involved across different brain imaging modalities remains unknown.

Previous conceptual studies have suggested that structural and functional networks of the brain involving cognitive control may be associated with psychological resilience. Parsons, Kruijt, and Fox (2016) proposed a neurocognitive model of psychological resilience that elucidates possible processing mechanisms. In this model [9], several important cognitive functions may contribute to the resilience process and allow people to cope with and react to stressful situations. Specifically, executive control involves top-down regulatory processes consisting of inhibitory control, working memory, and cognitive flexibility that are parts of the key functions in the prefrontal cortex (PFC) (see [10] for a review).

Kong et al. have reported on the role of PFC in psychological resilience in recent studies [3,8]. They employed a unimodal neuroimaging approach [i.e., resting-state functional magnetic resonance imaging (rfMRI)] to investigate the role of the PFC brain network in modulation between resilience and overall quality of life. Human brain neuroimaging techniques are widely used in neuroscience to improve our understanding of the brain’s structure and function and to identify biomarkers, especially for psychiatric diseases [7,11]. Likewise, this advanced imaging approach is suitable for revealing the mediation role of the brain’s structure and function in relation to psychological resilience [12]. Kong et al.’s [3,8] results demonstrated that the frontal cortex plays an important role in subjective wellbeing in healthy young adults. In line with this hypothesis of PFC involvement, we recently proposed a conceptual neurocognitive model, i.e., cognitive appraisal of resilience (CAR) model, based on the literature that elucidates potential brain mechanisms underlying human resilience [7]. The CAR model suggests that a disrupted frontal network of the brain impairs cognitive flexibility, leading to maladaptive behavioral outcomes. Specifically, the top-down process involves cognitive control and emotional regulation, which facilitates individuals to divert their attention from the pain experience induced by the perceived adverse events [13]. If this top-down regulatory process is disrupted, then the efficiency to divert attention is impaired, resulting in a malfunction of emotional regulation. According to two review articles [7,9], the structure and function of the brain regions that involve executive function, especially cognitive flexibility, may mostly be related to psychological resilience.

Despite that the aforementioned studies (e.g., [3,8]) have reported the role of PFC brain network in resilience and quality of life, none of these studies have examined the association between multimodal imaging properties and psychological resilience. Hence, the motive and novel aspect of the current study was to utilize a multimodal neuroimaging approach that combines datasets obtained with two or more unimodal imaging modalities (e.g., gray matter volume, white matter diffusion, and functional hemodynamic response data) rather than a single neuroimaging technique to examine the neural basis of psychological resilience. The advantage of a multimodal neuroimaging approach is to overcome each neuroimaging technique’s limitations [14]. The idea of applying multimodal measures is inspired by recent advances in brain imaging development, which suggest that different correlational measures in either structural or functional data could generate distinct “connectivity” between brain regions that are largely different between modalities [15]. This imaging approach to fuse different imaging modalities has become widely used in clinical research because it can provide a comprehensive understanding of the brain and its disorders [16]. Different imaging data types should be leveraged to extract complementary information. For example, rfMRI measures the hemodynamic response that is related to neural activity at resting state, structural MRI (sMRI) depicts different tissue types in each voxel of the brain (e.g., gray matter volume, GMV), and diffusion MRI (dMRI) provides information regarding the integrity of white matter tracts and structural connectivity [16].

Calhoun et al. (2009) developed a data-fusion process that utilizes multiple types of images simultaneously in order to take advantage of the cross-information from different unimodal image data [17]. In the present study, we employed the method of fusion independent component analysis (fusion ICA) [18] developed by Calhoun et al. (2006) [19]. The fused brain data includes volume-based morphometry (vbm) to represent the brain’s volumes, especially the gray matter’s volume [20]. Diffusion-tensor imaging (DTI) is used to represent the white matter integrity [21], and functional imaging based on the amplitude of low-frequency fluctuation (ALFF) is used to represent the regional intensity of spontaneous fluctuations in the BOLD signal [22]. This fusion ICA method and these types of multimodal imaging data have previously been reported successfully by our lab to examine the multimodal imaging fusion components and age-related differences in executive functions across the adult life span.

To address the issue of multimodal neuroimaging in relation to psychological resilience, this study used a Chinese version of the Resilience Scale for Adults (RSA) [23]. The original RSA is a self-report questionnaire that consists of 33 items for evaluating six dimensions: (1) perception of the self, (2) planned future, (3) social competence, (4) family cohesion, (5) social resources, and (6) structured style [23]. The Chinese version of the RSA consists of 29 items (removing 4 items from the original RSA) for evaluating five dimensions: (1) personal strength, (2) family cohesion, (3) social resources, (4) social skills, and (5) planned future. In particular, this study focused on the RSA’s subscale of personal strength [23,24] because previous research has indicated that resilience is associated with a personality trait pattern comprising maturity, responsibility, optimism, perseverance, cooperativeness, and persistence [25,26,27]. Personal strength reflects the individual characteristics that are crucial for predicting an individual’s reactions to negative life events. This study examines the neural correlates of psychological resilience by exploring the association between resilience’s subscale of personal strength and the outcome of the fusion ICA in multimodal neuroimaging data, which include information about the brain’s structural and functional properties. Based on previous conceptual brain models [7,9] and neuroimaging studies [3,8] on resilience, we speculated that psychological resilience, especially personal strength score [23,24], would associate with frontal-related networks derived from the fusion ICA. Specifically, the way people respond to adverse experience varied between individuals [28]. Review studies in the application of brain imaging to study resilience have reported the involvement of the prefrontal cortex in relation to resilience [7,29]. However, the evidence from these review studies to support the prefrontal cortex mostly relies on single imaging modalities (structural or functional). These findings lack integrated information from these imaging modalities to provide stronger arguments regarding the role of the prefrontal cortex in resilience. Moreover, whether or not an individual develops post-traumatic symptoms after experiencing a traumatic event is dependent on an individual’s resilience (i.e., intra-individual variation) [28]. Hence, this study bridges the gap between the involvement of the prefrontal cortex (fusion multi-imaging modalities approach) in a person’s resiliency (i.e., personal strength of resilience). Specifically, we hypothesized multiple frontal-associated regions related to emotion-regulation and cognitive control of subjective feelings of pain were involved. For example, participants who experienced war or sexual abuse without diagnosed traumatic symptoms have stronger activation in frontal regions such as the middle temporal gyrus and right anterior frontal regions than those who developed traumatic symptoms [30,31,32,33]. Furthermore, in the sexually abused group, those without developing post-traumatic symptoms exhibited stronger activation in the right hippocampus, inferior fusiform gyrus, supramarginal gyrus, and visual association cortex than those (especially women) who developed post-traumatic stress disorder (PTSD) [30,31,32,33]. These brain regions include frontal lobes for top-down regulation of negative affect as well as posterior lobes involving stimulus-driven visual attention to orient perception of perceived experiences [7]. This possibly suggests that there may be specific correlates of a person’s resilience or vulnerability for an individual’s reaction to negative events. To support this notion, we expect fusion multimodalities imaging components in frontal-associated regions that are reported from previous studies to be related to an individual’s resilience level as measured by a subscale of resilience (i.e., personal strength). This study would provide more integrated information to examine if frontal-associated regions are generally involved or specific to some imaging modalities. Furthermore, whether these associations are related to an individual’s resilience (i.e., personal strength of resilience scale).

## 2. Methods and Materials

### 2.1. Participants

We recruited 109 participants from Southern Taiwan through advertisements on the Internet and bulletin boards. There were 48 females and 61 males, and their mean age was 21.56 ± 1.88 years (standard error; *SD*) (range 19–30 years). All participants signed a written informed consent form that was approved by the Research Ethics Committee of National Cheng Kung University, Tainan, Taiwan, R.O.C. All participants were paid 1660 New Taiwan dollars (NTD) after completing the entire experiment.

### 2.2. Resilience Score Measurement

The Chinese version of the RSA consisting of 29 items [23] was used to measure an individual’s resilience. These items were scored using a seven-point semantic differential scale. There are five subscales (1) personal strength, (2) family cohesion, (3) social resources, (4) social skills, and (5) planned future. Only personal strength was included for subsequent analyses to investigate the questions of interest.

### 2.3. Image Acquisitions

We acquired magnetic resonance imaging (MRI) data by using a General Electronic (GE) MR750 3T scanner (GE Healthcare, Waukesha, WI, USA) in the Mind Research Imaging Center at National Cheng Kung University. T1-weighted structural images with high resolution were acquired with a fast-spoiled gradient-recalled echo sequence that consists of 166 axial slices (TR/TE/flip angle 7.6 ms/3.3 ms/12°; the field of view [FOV] 22.4 × 22.4 cm^2^; matrices 224 × 224; slice thickness 1 mm). The entire process lasted for 3 min 38 s.

The resting-state functional imaging data were collected using an interleaved T2 *-weighted gradient-echo planar imaging pulse sequence (TR/TE/flip angle = 2000 ms/30 ms/77°; matrices = 64 × 64; FOV = 22 × 22 cm^2^; slice thickness = 4 mm; voxel size = 3.4375 × 3.4375 × 4 mm). A total of 245 volumes were acquired which covered the entire brain of each participant. The first five dummy scans were discarded to reduce equilibrium effects on T1 images. During the resting-state functional scans, the participants were instructed to remain awake with their eyes open and fixate on a white cross shown on a screen. The total scanning time lasted for 8 min and 10 s per participant (i.e., [number of samples + number of dummy scans] × TR = [240 + 5] × 2 = 490 s).

The diffusion tensor imaging (DTI) data were obtained with a spin-echo-echo planar sequence (TR/TE = 5500 ms/62–64 ms; 50 directions with b = 1000 s/mm^2^; 100 × 100 matrices; slice thickness = 2.5 mm; voxel size = 2.5 × 2.5 × 2.5 mm; the number of slices = 50; FOV = 25 cm; NEX = 3). Reverse DTI was also acquired for top-up correction during the preprocessing steps. The acquisition parameters of the reverse DTI were identical to those of the DTI, except that only six directions were acquired to prevent the participant from getting exhausted.

### 2.4. Imaging Quality Control

The imaging quality control was based on head motion parameters and framewise displacement (FD). The screening criteria included that the participants’ maximum head motion did not exceed 2.5 mm, and the mean FD did not exceed 0.25. A visual inspection of all images was performed after the normalization and co-registration steps to ensure there was no bad warping.

### 2.5. Image Preprocessing

#### 2.5.1. Structural MRI (sMRI)

To retrieve sMRI data, we performed the following analysis steps. First, structural images were extracted from the brain by the BET function implemented in FSL [34]. The -N option was chosen because the image includes much of the neck. Voxel-based morphometry (VBM) [20] was used to characterize an individual’s brain structural differences. Second, tissue-type segmentation was carried out using FASTv4.0 [35]. The resulting grey-matter partial-volume images were then aligned to the GM ICBM-152 template [36] using the non-linear registration tool FNIRT [37].

The resulting images were averaged to create a study-specific template, in which the native grey matter images were then non-linearly re-registered. Next, we multiplied the registered partial volume images of all participants by using the Jacobian of the warp field (modulation) to correct for local expansion [38]. The modulated segmented images were finally resliced to 91 × 109 × 91 matrices with a voxel size of 2 × 2 × 2 mm and smoothed using an isotropic Gaussian kernel with a sigma of the full-width at half-maximum (FWHM) of 8 mm.

#### 2.5.2. Resting-State Functional MRI (rfMRI)

We used the CONN toolbox 18a (www.nitrc.org/projects/conn) and SPM 12 (http://www.fil.ion.ucl.ac.uk/spm) in Matlab (The MathWorks, Inc., Natick, MA, USA) to preprocess the functional images. The preprocessing protocol was modified from Geerligs and Tsvetanov’s study [39]. The first step consisted of slice timing, realignment, normalization (using a T1 image to register to standard space), and smoothing with an 8-mm Gaussian kernel. In addition, images were then resliced into the size of 2 × 2 × 2 mm, resulting in a data cube of 91 × 109 × 91 voxels. The second step was to calculate nuisance covariates (R), including movement parameters (translations along the x, y, and z axes and rotations along with three directions: roll, yaw, and pitch), white matter (WM) signals, and cerebral spinal fluid (CSF) signals [40]. The third step was to regress out bad frames at the subject level which were detected by “head motion censoring” and [R R^2^ R_t-1_ R^2^_t-1_], where t and t-1 refer to the current and immediately preceding time point, respectively [41]. The final step was to apply a band-pass filter with a range of 0.008–0.1 Hz to nuisance covariates and fMRI data simultaneously. For the rfMRI, we further extracted the voxel-wise mean amplitude of low-frequency fluctuations (ALFF; [22]) to generate a map for each participant. The ALFF was computed with the fast Fourier transform (FFT) on time series of each voxel, and then taking the square root of the power spectrum to obtain the amplitudes, which were further averaged in the range of 0.01–0.08 Hz (see [22] for details).

#### 2.5.3. Diffusion MRI (dMRI)

All processing and analyses of the diffusion-weighted imaging (DWI) data were computed by FMRIB Software Library (FSL v5.0.9; www.fmrib.ox.ac.uk/fsl [20]). The DWIs were first converted from the DICOM format to the NIFTI format via the MRIcron dcm2nii tool (https://www.nitrc.org/projects/mricron/). TOPUP [20,42] and EDDY [43] were used to clean the DWIs of artifacts caused by susceptibility-induced distortions, eddy currents, and head motion. The b0 image was extracted from the concatenated data with which non-brain tissue was removed via the FMRIB BET tool [44] to create a brain mask for subsequent analyses.

DTIFIT [45] was applied to fit a tensor model (i.e., diffusion tensor imaging, DTI) at each voxel of the data [16] to derive measurements of the fractional anisotropy (FA), mean diffusivity (MD), and radial diffusivity (RD). Tract-based spatial statistics (TBSS) in FSL [21,46] was used to perform tract-based investigations of the DTI measurements. The FA images were slightly eroded, and the end slices were zeroed to remove outliers from the diffusion tensor fitting. All FA images for each participant were then non-linearly aligned to an FMRIB58_FA standard-space image (1 × 1 × 1 mm).

Next, the group-mean FA image was performed by a skeletonization procedure, and the result was set at a threshold of FA > 0.2 to identify the areas that were most likely to belong to white-matter tracts of non-trivial size [47]. For each participant, the FA data were projected onto the mean FA skeleton. Individual skeletonized FA images were resliced to a final 91 × 109 × 91 matrices with the voxel size of 2 × 2 × 2 mm. Finally, the images were smoothed with an 8-mm^3^ FWHM Gaussian kernel. This process was then repeated for MD and RD images using the tbss_non_FA function.

### 2.6. Joint ICA Analysis

Preprocessed data (sMRI, rfMRI, and dMRI) were fused in Matlab using the Fusion ICA Toolbox (FIT, [48,49] http://mialab.mrn.org/software/fit/index.html). The fusion approach was identical to that used in a previous study by our group [50,51]. After feature extraction, the 3D image of each participant was reshaped into a single row and stacked individually to form a matrix with dimensions of 109 × [number of voxels] for each imaging modality. Because there were different scales for the three imaging modalities, the feature matrix was normalized to yield the same average sum-of-squares that were computed across all participants and all voxels for each imaging modality.

After normalization, the data were further processed by dimension reduction, followed by joint ICA, and component selection. The component number was estimated using the modified minimum description length (MDL) criteria [52]. We chose MDL = 36 for the following analysis.

The data dimensionality was reduced by principal component analysis. The infomax algorithm [53] decomposed the reduced feature matrix to subject-specific mixing (loading) parameters and maximally independent component images. We used ICASSO to run the ICA algorithm 5 times and produce different estimated components for each run. The components were then collected by clustering them based on the absolute value of the correlation between squared source estimates.

The sMRI, rfMRI, and dMRI spatial maps were visualized by transforming each component into a Z map, which was divided by its standard deviation (SD) across all voxels. The use of Z-scores involves linearly transformed data values and standardized distributions, so that each component has a mean of zero with SD of one. A negative value simply indicated that the original value in the map was below the mean before it was standardized, therefore, we could only assume its relative place in the distribution [54]. The spatial maps were thresholded with |Z| > 2.5.

To display rfMRI and sMRI maps, each component’s clusters were first converted from MNI coordinates to Talairach coordinates and then entered into a database to provide anatomic and functional labels for the right (R) and left (L) hemispheres. To display the dMRI clusters, we used the Johns Hopkins white-matter tractography atlas provided in FSL as ROI mask to identify 18 DTI tracts [55]. These tract masks were used to mask the RD map and calculate the clusters’ percentages in tracts. All these clusters have also been transformed into a binary mask. After excluding the brainstem and cerebellum regions, we used these masks to extract signals from the original features of the participant.

## 3. Statistics for Correlation

Bayesian partial correlation was performed in R (version 3.0.2; R Foundation for Statistical Computing, http://www.R-project.org) using BayesMed toolboxes (https://CRAN.R-project.org/package=BayesMed) to test for the association between participants’ RSA_p and the joint ICA components’ mixing coefficient while controlling for gender as a covariate of no interest. Bayes factors [56,57] may be interpreted as proportional evidence for the presence or absence of an effect. For instance, a BF_10_ of 5 is considered as the data that are 5 times more likely to occur under the alternative hypothesis than under the null-hypothesis. The Leave-One-Out cross-validations were applied to estimate the performance between actual RSA_p and predicted RSA_p.

### 3.1. Multiple Regression Model for Predicting RSA Scores

We extracted signals from every participant’s fusion imaging components (both positive and negative values across all imaging modalities). Six measures were defined as independent variables. We then used multiple regression to test the relationship between these independent variables and resilient measurements (i.e., RSA scores). The Bayesian version of these tests was also performed.

### 3.2. Leave-One-Out Cross-Validation

In this step, we extracted the degree of freedoms and R-squared from each linear regression model, and set the alpha as 0.05. Each prediction from a component was measured with Pearson’s correlation to estimate if the predicted RSA_p was correlated with the actual RSA_p. Leave-one-out cross-validation was applied for the model training in each individual. The training was performed using n-1 participants for predicting the leaved-out participant’s performance [58]. To assess the significance between actual RSA_p and predicted RSA_p generated from leave-one-out cross-validation, the non-parametric *p*-value using a permutation test with “5000” iterations with an alpha of 0.05 as significance. This resulted in a null distribution composed of “5000” *r* values, and the *p* values were calculated by the percentile where the generated *r* values were equal to or larger than the null values [59]. Only the fusion component that was significant in the permutation test and yielded a positive correlation with resilience score could be retained, which indicated a robust and successful prediction.

## 4. Results

### 4.1. Neuropsychological Test Scores: BDI-II and RSA

The mean BDI-II score for participants was 7.78 ± 6.52 (*SD*). The mean RSA score was 146.51 ± 22.27. The subscale scores of the RSA are (1) 28.22 ± 6.48 for personal strength (RSA_p), (2) 35.41 ± 7.62 for family cohesion (RSA_f), (3) 43.98 ± 7.96 for social resources (RSA_sr), (4) 20.04 ± 5.13 for social skills (RSA_s), and (5) 18.85 ± 5.49 for planned future (RSA_fu).

### 4.2. Independent Components (ICs)

**Figure 1:**
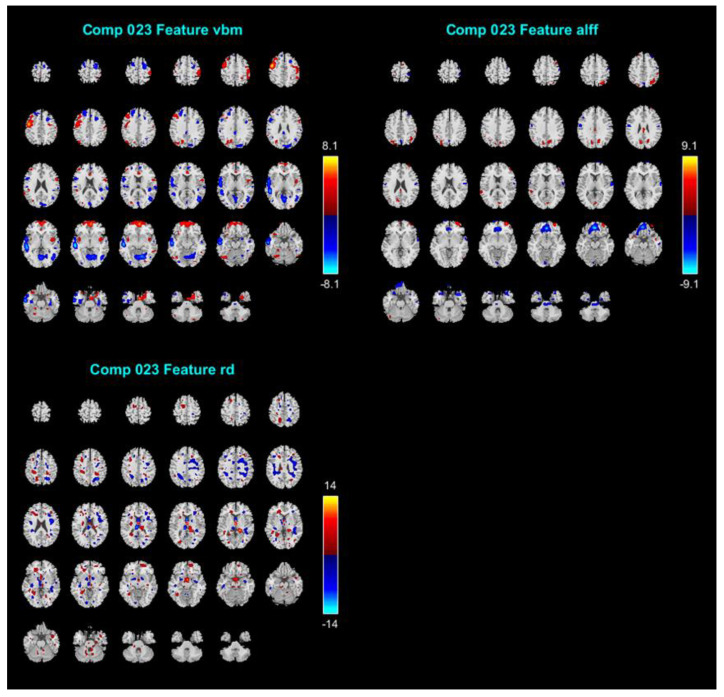
The results of IC #23 in relation to the RSA (Resilience Scale for Adults) subscale of personal strength (RSA_p). The spatial maps shown in this figure were transformed into Z values visualized at |Z| > 2.5. ICs = independent components.

Of the 38 components, we discarded 14 ICs because their stability was less than 0.8, as well as another 4 ICs because they contained obvious artifacts including sharp edges around the brain boundary or within the cerebrospinal fluid (CSF) region. The remaining 20 ICs’ mixing coefficients were retrieved to be correlated with the subscale’s score of RSA using Person’s *r* and the Bayesian correlations [60]. The mixing coefficients represent the relative degree to which an individual participant contributes to the joint component. The results are shown in Table 1. Only IC#23’s mixing coefficients yielded a significant correlation with RSA_p (*r* = 0.269; BF_10_ = 7.554). Hence, we report the results for IC #23 in relation to the resilience measure in detail (see Figure 1). The spatial maps shown in Figure 1 were transformed into Z values visualized at |Z| > 2.5. The volume of identified voxels in each area is provided in cubic centimeters (cm^3^).

### 4.3. IC#23 Different Spatial Maps Associated with Psychological Resilience (RSA_p)

Figure 1 displays IC#23′s spatial maps of GMV (e.g., VBM), ALFF, and RD tensor-based WM tracts. For GMV map, the negative contributing regions (i.e., significantly negative correlations) were mainly in the temporal lobe (superior, middle, and inferior temporal gyrus), and secondly in lingual gyrus, precentral, superior frontal gyrus, middle frontal gyrus, inferior frontal gyrus, medial frontal gyrus, postcentral gyrus, middle occipital gyrus, insula, parahippocampal gyrus and the cuneus. Whereas the positive contributing regions included precentral gyrus, postcentral gyrus, frontal lobe (superior, middle, inferior, and medial), insula, inferior parietal lobule, superior temporal gyrus, precuneus, anterior cingulate, middle occipital gyrus, fusiform and angular gyrus. For the ALFF map, the negative contributing regions are mainly in the medial frontal gyrus, precentral gyrus and superior temporal lobe, and were in the minority in the anterior cingulate, orbital gyrus, superior frontal gyrus, middle frontal gyrus and postcentral gyrus, inferior frontal gyrus, and lingual gyrus. Whereas the positive contributing regions are mainly in the superior frontal gyrus, middle frontal gyrus, precuneus, cuneus, cingulate gyrus, superior temporal gyrus, and superior parietal lobule. For WM tracts map, the negative contributing clusters are mainly in the SLF and IFF.

### 4.4. Interaction of Multi Modalities Among rfMRI, sMRI, and dMRI of IC#23 and Its Association with RSA_p

**Figure 2:**
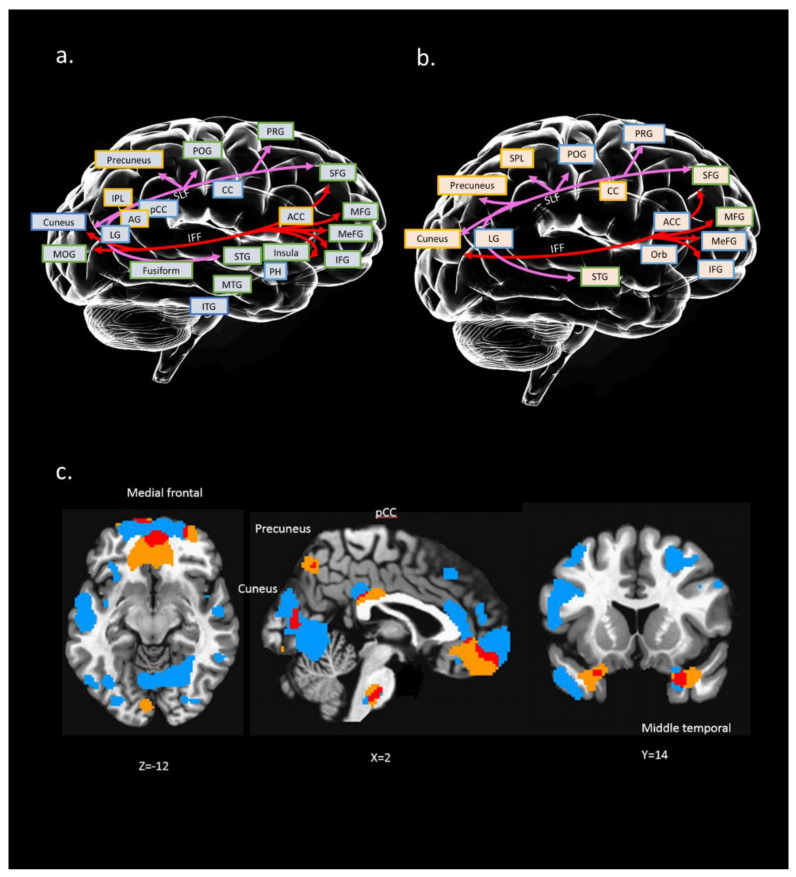
Conceptual illustration to present possible relation between brain regions (structural and functional) and connection paths [white matter (WM) tracts]: (**a**) interaction between WM tracts and GM region of IC#23; (**b**) interaction between WM tracts and ALFF regions of IC#23. A colored square with a yellow edge indicates positive clusters in this region. A colored square with a blue edge indicates negative clusters in this region. Colored square with green edge indicates both positive and negative clusters in this region; (**c**) overlapping (marked in red) between IC #23’s GM (marked in blue) and ALFF regions (marked in yellow); x, y, z = 2, 14, −12. Distinct WM tracts were marked with different colors. PRG: precentral gyrus; POG: postcentral gyrus; MFG: middle frontal gyrus; MeFG: medial frontal gyrus; IFG: inferior frontal gyrus; CC: cingulate cortex; ACC: anterior cingulate; pCC: posterior cingulate cortex; PH: parahippocampal gyrus; STG: superior temporal gyrus; MTG: middle temporal gyrus; ITG: inferior temporal gyrus; IPL: inferior parietal gyrus; LG: lingual gyrus; MOG: middle occipital gyrus; Orb: orbital gyrus; AG: angular gyrus.

Figure 2 further summarizes the main results of spatial maps across different neuroimaging modalities (i.e., rfMRI, sMRI, and dMRI). The purpose of this approach is to measure the interaction of the results with (a) known WM tracts for dMRI and (b) known brain regions for rfMRI and sMRI. Furthermore, we only present the significant results of ALFF and GM regions that are near to the WM tracts.

We found that the neural substrates of IC#23 cover a wide range of brain structures and functions, including the frontal region (e.g., medial frontal, superior frontal), temporal region (e.g., superior temporal, middle temporal, inferior temporal), parietal region (e.g., superior parietal, inferior parietal), occipital region (e.g., middle occipital, cuneus, precuneus), and even the subcortical region (e.g., cingulate gyrus, parahippocampal gyrus) (see Figure 2). Also, two tracts in IC#23 were SLF and IFF, which connected the frontal, occipital, parietal, and temporal lobes.

### 4.5. Multiple Regression Model of IC#23 for Predicting RSA_p Scores

We further examined whether the observed association between IC#23 and RSA_p can be used to predict actual behavioral scores for resilient individuals. To do so, we performed a multiple linear regression analysis to examine how these IC#23′s imaging measures were associated with RSA_p scores (multiple R^2^ = 0.14, *p*-value = 0.003; see Table 2). The results showed that IC#23′s imaging measures were associated with subscales of RSA_p scores. Specifically, IC#23′s positive and negative clusters were defined as ROI, and we extracted the signal from every subject’s original data. These six measurements were tested for their linear relationship with the outcome RSA_p, but only four of them satisfied the criterion. ALFF_positive, GM_negative, GM_positive, and RD_negative were used to predict RSA_p as in the formula below:

RSA_p~ALFF_positive + GM_negative + GM_positive + RD_negative

**Figure 3:**
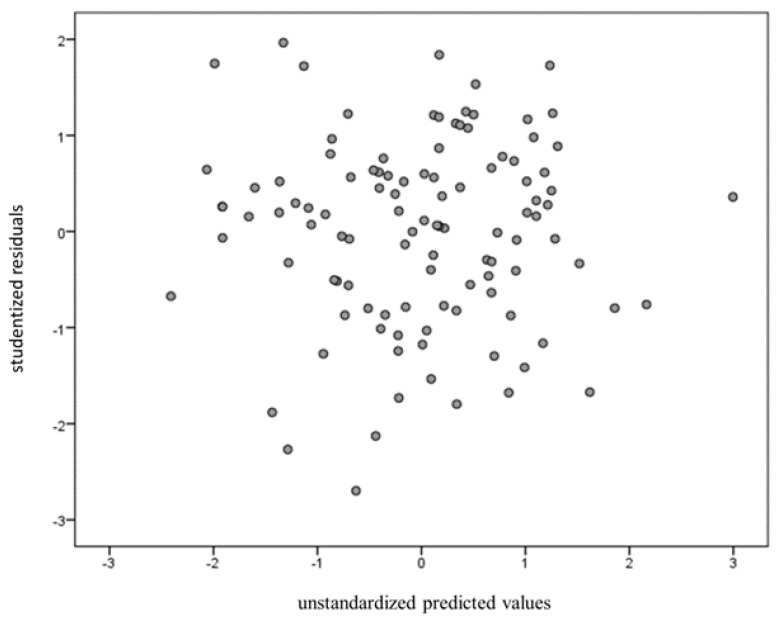
Studentized residuals were plotted against the unstandardized predicted values to examine homoscedasticity.

After controlling for gender as a covariate, the change in R-squared was 0.142. BF_10_ was 13.768, which indicates strong evidence for H1. Multicollinearity was tested on all four independent variables’ variance inflation factors (VIFs) less than 10 [61]. Durbin-Watson values were also calculated to test for independence. The result was 2.06, which is higher than the dU value of 1.62. The Shapiro-Wilk test was used to test for normality but showed no significant normal distribution. Finally, studentized residuals were plotted against the unstandardized predicted values to examine the homoscedasticity (Figure 3).

### 4.6. Leave-One-Out Cross-Validation

The result of cross-validation shows significant association performance between actual RSA_p and predicted RSA_p (Pearson’s *r* = 0.253; permutation *p* value = 0.002).

## 5. Discussion

The aim of this study was to identify the multimodality neural features that are associated with psychological resilience. Specifically, a fusion joint ICA approach with different modalities (sMRI, rfMRI, and dMRI) was used to investigate whether multimodal imaging measures are associated with self-reported RSA scores, especially the personality subscale (i.e., RSA_p). Furthermore, we examined whether the observed association can be used to predict actual behavioral scores for resilient individuals by using a multiple regression model with a permutation test to validate the RSA_p scores prediction.

The results of the jICA analysis showed that one fusion multimodal imaging component (i.e., IC#23) spanning over the frontal-associated regions was significantly associated with the RSA_p scores. These frontal-associated regions included the medial prefrontal cortex (mPFC) [62], anterior cingulate cortex (ACC) [63,64], amygdala [65], and hippocampus [65,66], which have been evident in research on neuroimaging correlates of resilience studies [65,67]. However, findings from brain imaging studies reported regions related to resilience are not limited to these regions. For example, participants who experienced war or sexual abuse without diagnosed traumatic symptoms have stronger activation in frontal regions such as the middle temporal gyrus, and right anterior frontal regions than those who developed traumatic symptoms [30,31,32,33]. Furthermore, in the sexually abused group, those without developing post-traumatic symptoms exhibited stronger activation in the right hippocampus, inferior fusiform gyrus, supramarginal gyrus, and visual association cortex than those who developed PTSD [30,31,32,33]. These regions included frontal regions for top-down regulation of negative affect as well as stimulus-driven visual attention to orient perception of perceived experiences [7]. This possibly suggests that there may be specific correlates of a person’s resilience or vulnerability for an individual’s reaction to negative events. The regression model of this study revealed that the neural substrates covered in IC#23 can indeed predict the RSA_p scores. These findings suggest that the dynamic interactions of the brain among frontal-associated networks are related to an individual’s resilience strength in response to negative life events.

The finding that IC#23 is associated with RSA_p appears to support our prior hypothesis that cognitive control brain networks (e.g., frontal-related brain regions) serve as multimodal neural networks for resilience strength in one’s personality [7,9]. The neural substrates of IC#23 cover a wide range of brain structures (reflected on sMRI GMV) and functions (reflected on rfMRI ALFF), including the frontal region (e.g., medial frontal, superior frontal), temporal region (e.g., superior temporal, middle temporal, inferior temporal), parietal region (e.g., superior parietal, inferior parietal), occipital region (e.g., middle occipital, cuneus, precuneus), and even the subcortical region (e.g., cingulate gyrus, parahippocampal gyrus) (see Figure 2). Specifically, in IC#23, overlapped imaging modalities mainly converged in the anterior part of the frontal lobe, which is associated with resilience, especially the personal strength aspect of the RSA score. One prominent feature of the novel fusion approach used in this study is to reveal associations that cannot be discovered by separate multimodal analyses [16,68]. The current results clearly suggest that alterations in the brain structure can be associated with changes in functional brain activation, connecting brain regions that interact in imaging modalities. Therefore, multimodal fusion proves to be a powerful tool to reveal this association in connecting interaction among imaging modalities overlapped in frontal-associated regions with personal strength of resilience scale. This finding may suggest that the anterior regions of the frontal cortex are associated with personal strength of resilience to the negative effect of adverse experiences.

The current findings suggest that interaction between frontal-related networks (i.e., cognitive control networks) regulates individuals’ scoring high on RSA_p who are psychologically healthier, better adjusted, and more resilient in the face of adverse events (i.e., an individual’s capacity to positively cope with a negative adverse event for successful adaption). An important study on the relationship between RSA scales and other measures (e.g., different types of intelligence and personality) suggested that the RSA subscale of personal strength is the most related to stabilizing emotional responses [24]. This view can be supported by a recent study [69] using task-related functional MRI to investigate the BOLD signals changes when induced emotional responses to negative pictures in women with and without the development of PTSD. Their results found that the ability to upregulate emotional responses in prefrontal regions to negative stimuli may be a protective factor in the face of trauma exposure and associated with resilience. Therefore, the ability to regulate emotions to maintain emotional stability can be seen as a key factor for an individual’s resilience to conquer adverse experiences.

In line with these findings, the results echo a recent review on the potential mechanism of psychological resilience, showing that emotional regulation plays an important role in relation to an individual’s resilience level [7]. Consistent with this hypothesis, the brain regions covered in IC#23, such as the PFC, temporal, and subcortical regions, have also been evidenced as being important for emotion regulation [70]. Therefore, the current results are in line with the idea that an individual’s resilience strength is related to that individual’s ability to maintain and stabilize the emotional response.

As mentioned, the major overlapped brain regions across imaging modalities for IC#23 were in the anterior part of the PFC, specifically in the medial frontal regions. These findings of the brain structure and function in the medial frontal cortex, anterior cingulate, and parahippocampal appear to be consistent with animal studies on resilience. These studies reported that certain brain regions are involved in perceiving adverse events, such as the medial PFC (mPFC), anterior cingulate cortex (ACC), amygdala, and hippocampus [7]. In addition, the findings are also consistent with human research in which the ventromedial PFC has been linked to the reward valuation system [71] and has been suggested to be involved in experiencing safety during otherwise threatening situations [72,73]. Thus, this area serves as a potential candidate for involvement in positive appraisal during adverse situations.

The medial frontal lobe overlaps with the orbitofrontal cortex, which is involved in encoding pain or pleasure [74,75,76,77,78,79,80]. This is in line with behavioral studies reporting a positive correlation between psychological resilience and reward experience [81,82,83]. The role of the medial frontal lobe has been extensively discussed in a study relating to the cognitive mechanisms underlying resilience [7]. These findings support the hypothesis that the PFC is involved in constructing reappraisal strategies that can modulate activity in multiple cognition–emotion processing systems. Furthermore, this region is also considered to be a critical node of brain networks underlying emotion regulation [84,85,86,87,88], which corresponds to the view that “psychologically resilient individuals are emotionally intelligent” [89,90]. Thus, this brain region in association with resilience may be involved in processing the reward value of different stimuli and regulating daily emotions.

The current findings show that it is not only the medial frontal lobe that is associated with resilience, but also the posterior regions and two WM tracts that connect between the anterior and the posterior regions. Specifically, two major WM tracts (reflected on dMRI RD) are covered in this component: the SLF and IFF in the right hemisphere, which connect the superior frontal gyrus, cuneus, and middle occipital gyri (see Figure 2). For example, Puglisi et al. (2019) investigated mimicked frontal lobe lesions by directly stimulating patients with frontal right hemisphere glioma during an intraoperative Stoop test [91]. They observed that there were more performance errors produced by these stimulated patients. This phenomenon suggested that there is a key component of cognitive control in the right hemisphere, in which the right IFG and its connections with the striatum may be fundamental for this function. Rowe et al. (2005) suggested that the effective connectivity between the PFC and the posterior cortex changes as subjects switch between performing different tasks [92]. These findings may help to explain our results of frontal-associated regions covering cognitive control in a visual-related network as adverse events are visually perceived and internalized by regulating cognitive-emotional processes. Therefore, the current results suggest that frontal-occipital networks play an important role in psychological resilience.

In our review article [7], we proposed a CAR model in which two foundations make resilience a dynamic developmental process: top-down cognitive control (e.g., goal-directed) and bottom-up processing (e.g., stimulus-driven). Top-down processing refers to the active inference that is driven by prediction, while bottom-up processing refers to processing that is built up from passive, perceived, external sensory information. Overall, frontal brain circuitry serves to control cognition and emotion by connecting the perceived adverse experience of subjective suffered feelings. Thus, it is not surprising to observe a network that connects frontal and occipital regions via the SLF and IFF in association with psychological resilience. Based on the model, we may reason that the frontal lobe regions involve processes that actively regulate human resilience by interacting with the posterior brain regions that perceive emotion and pain from adverse events. Such interaction facilitates the processes of reappraisal of adverse events and the positive adaptation to subsequent events.

Before closing, there are some limitations to the current study that require discussion. First, confounding variables (e.g., genetic, epigenetic, brain structure, brain function, neurochemical, physiological, developmental, demographic, cultural, economic, social, and psychological variables [2,3,4,5,6,7]) that might weaken the validity of resilience measurements. Future study is warranted to incorporate more other variables for studying gene-environment interaction in combination with neuroinformatic in order to improve the predictivity of resilience. For example, some researchers have indicated that in some cases [93], using combined genetic and fMRI data might achieve better classification accuracy than using alone [94,95]. This indicates that genetic and brain function represent different but partially complementary aspects [18].

Second, in this study, we used the RSA’s subscale of personal strength to evaluate resilience, yet there are other types of self-reported scales available to quantify individuals’ psychological resilience, such as Connor Davidson Resilience Scale (CD-RISC) [96] and, Brief Resilience Scale (BRS) [97]. Therefore, future studies are needed to directly compare if other scales can obtain similar results. Despite these available instruments that can quantify resilience, they are nevertheless subjective relying heavily on self-report information, which warrants future research.

Finally, although we analyzed FA, RD, and MD for DTI, we mainly observed that RD of IC#23 was significantly associated with resilience. RD is typically seen as measuring myelin integrity, and this imaging property may be affected by the presence of crossing fibers and residual misalignment [98]. Moreover, a study [99] has shown that RD is more sensitive than other DTI measures (e.g., FA, MD) to the demyelination process (e.g., [100]), which is strongly involved in age-related brain deterioration [101,102,103,104,105,106,107]. Specifically, (de)myelination as measured by RD has shown a relation to processing speed [108] in children [109] and the elderly [110]. Therefore, the current result showing that only RD was sensitive to resilience suggests that the processing efficacy (as reflected in myelination) plays a more important role.

## 6. Conclusions

Our study used a state-of-the-art fusion approach to combine different brain imaging modalities in relation to resilience scores. The results revealed a fusion component associated with the subscale’s score of resilience. Multiple regression models of the fusion component for predicting resilience scores suggested that the component explained most of the individual variances in the specific RSA scale that pertain to personal strength. These results suggested that interaction among multiple imaging modalities in frontal-associated regions is likely to serve as a multimodal neural network for resilience strength at the individual level.

## Figures and Tables

**Table 1:** The correlation between independent components (ICs) and the RSA (Resilience Scale for Adults) subscale of personal strength (RSA_p).

ICs	*r*	BF_10_	95%CI Upper	95%CI Lower
IC #2	−0.008	0.118	−0.185	0.169
IC #4	0.090	0.185	−0.097	0.276
IC #5	0.068	0.152	−0.118	0.258
IC #7	−0.088	0.186	−0.270	0.092
IC #8	0.045	0.132	−0.146	0.234
IC #9	−0.046	0.133	−0.232	0.139
IC #10	−0.054	0.141	−0.233	0.124
IC #11	0.095	0.200	−0.091	0.280
IC #12	−0.117	0.257	−0.302	0.069
IC #13	−0.044	0.132	−0.229	0.139
IC #14	−0.065	0.148	−0.252	0.120
IC #15	−0.015	0.119	−0.201	0.175
IC #16	0.154	0.443	−0.031	0.342
IC #17	−0.037	0.127	−0.224	0.150
IC #18	0.102	0.214	−0.084	0.290
IC #19	−0.069	0.153	−0.255	0.119
IC #20	0.018	0.120	−0.208	0.169
IC #21	−0.025	0.122	−0.214	0.164
IC #22	−0.030	0.124	−0.217	0.157
IC #23	0.269	7.554	0.090	0.451

**Table 2:** The results of general regression for four neural variables and the RSA (Resilience Scale for Adults) subscale of personal strength (RSA_p).

	Coefficients	Std Error	*t*-Value	*p*
(Intercept)	−0.00	0.09	−0.00	0.99
ALFF_positive	−0.20	0.11	−1.76	0.08
GM_negative	0.16	0.10	1.55	0.12
GM_positive	−0.03	0.10	−0.34	0.74
RD_negative	0.18	0.09	1.90	0.06

Note: ALFF: amplitude of low-frequency fluctuations; GM: gray matter; RD: radial diffusivity.

## Data Availability

The data presented in this study are available on request from the corresponding author. The data are not publicly available due to the ethical concern.
